# The Union Dental Convention

**Published:** 1890-08

**Authors:** 


### THE UNION DENTAL CONVENTION.

It will be observed by the card on another page that the profession in New England propose to combine fourteen different societies in one meeting on October 28-31, 1890. This union meeting promises to be one of the largest as well as one of the most valuable held in that section. It truly represents New England, and no doubt will have its influence in leading to other sectional organizations to take the place of isolated society effort.

They propose to make the exhibition of "articles, instruments, and materials of use in dentistry" not only full, but instructive, including in it a department of dental journals. This idea, in its proposed extent, is, it is believed, novel in dental conventions, and should be of great value in educating many to the importance as well as to the extent of this branch of dental work.

